# Dyskerin depletion increases VEGF mRNA internal ribosome entry site-mediated translation

**DOI:** 10.1093/nar/gkt587

**Published:** 2013-07-01

**Authors:** Laura Rocchi, Annalisa Pacilli, Rajni Sethi, Marianna Penzo, Robert J. Schneider, Davide Treré, Maurizio Brigotti, Lorenzo Montanaro

**Affiliations:** ^1^Department of Experimental, Diagnostic and Specialty Medicine, Alma Mater Studiorum-Universita’ di Bologna, Bologna 40126, Italy, ^2^Centro Interdipartimentale di Ricerche sul Cancro ‘Giorgio Prodi’-CIRC, Alma Mater Studiorum-Universita’ di Bologna 40138, Italy and ^3^Department of Microbiology, New York University School of Medicine, New York, NY 10016, USA

## Abstract

Dyskerin is a nucleolar protein encoded by the DKC1 gene that (i) stabilizes the RNA component of the telomerase complex, and (ii) drives the site-specific pseudouridilation of rRNA. It is known that the partial lack of dyskerin function causes a defect in the translation of a subgroup of mRNAs containing internal ribosome entry site (IRES) elements such as those encoding for the tumor suppressors p27 and p53. In this study, we aimed to analyze what is the effect of the lack of dyskerin on the IRES-mediated translation of mRNAs encoding for vascular endothelial growth factor (VEGF). We transiently reduced dyskerin expression and measured the levels of the IRES-mediated translation of the mRNA encoding for VEGF *in vitro* in transformed and primary cells. We demonstrated a significant increase in the VEGF IRES-mediated translation after dyskerin knock-down. This translational modulation induces an increase in VEGF production in the absence of a significant upregulation in VEGF mRNA levels. The analysis of a list of viral and cellular IRESs indicated that dyskerin depletion can differentially affect IRES-mediated translation. These results indicate for the first time that dyskerin inhibition can upregulate the IRES translation initiation of specific mRNAs.

## INTRODUCTION

Dyskerin is a nucleolar protein encoded by the *DKC1* gene (1) with two major functions. First, it participates in the site-specific conversion of rRNA uridines into pseudouridines, a process known as pseudouridylation (2). Ribosomal RNA pseudouridylation is necessary for proper pre-rRNA maturation and involves the activity/presence of three other proteins (GAR1, NOP10 and NHP2) and a number of specific small nucleolar RNAs containing a Hinge-ACA box (H/ACA snoRNA), which guide the protein core to specific uridine residues to be modified (3). Secondly, dyskerin stabilizes the telomerase RNA component, thus inducing the proper activity of the telomerase complex (4). Mutations in the *DKC1* gene cause the rare multisystemic syndrome ‘X-linked dyskeratosis congenita’ (X-DC), a disease characterized by a progressive failure of proliferating tissues (e.g. bone marrow and skin) associated with an increased risk of developing tumors (5,6). In the *DKC1* hypomorphic mouse, a partial lack of dyskerin function is associated with the increased development of different tumor types, including breast carcinomas (7). The observations that, in this model, there is both an impairment in rRNA uridine modifications and an increase in cancer incidence are already made in early generations, when telomeres are still long. This suggested the hypothesis that the alteration in ribosome integrity might determine an alteration in the translation of proteins that are important in tumor development, thus contributing to cancer development (8). In keeping with this, it is known that mutations of the *DKC1* gene cause a defect in the translation of a subgroup of mRNAs containing internal ribosome entry site (IRES) elements such as those encoding for the anti-apoptotic factors Bcl-XL and XIAP, as well as for the tumor suppressors p27 and p53 (9–11). The defect in the protein synthesis described in cells originated from X-DC patients and DKC1 hypomorphic mice has also been observed in a sub-set of human breast carcinomas characterized by a strong reduction in DKC1 expression and function (12). This indicates that the qualitative defects in ribosome biogenesis may contribute to cancer development not only in this rare inherited disorder, but also in tumors registered in the general population.

Angiogenesis, the process by which new blood vessels are formed, is required for the survival of many solid tumors, including breast carcinomas (13). Angiogenesis is driven by pro-angiogenic factors produced and released by both cancer and stromal cells. One of the well-characterized factors is the vascular endothelial growth factor (VEGF). VEGF has many functions: it acts as a mitogen for endothelial cell proliferation and as a survival factor that prevents endothelial cell apoptosis; it stimulates vascular permeability and promotes the recruitment and the differentiation of endothelial cell progenitors (14). In the unusually long VEGF mRNA 5′UTR, there are secondary structures with an IRES activity, which makes its translation possible when the total protein synthesis is reduced, such as when cells are exposed to low oxygen and nutrient concentrations (15). It has recently been demonstrated that VEGF can stimulate the growth of breast cancer cells directly (16,17).

In the present study, we investigated how the VEGF-IRES–mediated translation might be modulated by the lack of dyskerin in human breast epithelial cells. Our results demonstrate that dyskerin knock-down (KD) increases VEGF levels through a stimulation in IRES-dependent translation. These results suggest that dyskerin might have different effects in the translation of different IRES elements, thus providing new insights in the mechanisms of IRES-mediated translation initiation.

## MATERIALS AND METHODS

### Cells and reagents

All the cells used were obtained from ATCC. Human breast cancer–derived cell lines MCF7 and MDA-MB231 were cultured in a monolayer at 37°C in a humidified atmosphere containing 5% CO_2_. MCF7 and MDA-MB 231 were grown in Roswell Park Memorial Institute 1640 medium (Euroclone) and Dulbecco's modified Eagle's medium (Euroclone), respectively, supplemented with 10% of fetal bovine serum (Euroclone), 2 mM l-glutamine (Euroclone), 100 U/ml penicillin and 100 mg/ml streptomycin (Euroclone).

MCF7 were grown both in normal oxygen concentration and inside a hypoxia chamber at 1% oxygen. After 72 h of hypoxia, they were trypsinized and processed as described above. When indicated, cells were treated with 15 or 100 nM PTC299 (NCI thesaurus number C91100—PTC Therapeutics) for 48 or 72 h.

### RNA interference

For transient DKC1 KD, double-stranded short interfering RNAs (siRNAs) and an appropriate control were obtained from Invitrogen. DKC1 RNAi was performed with three pooled siRNA oligonucleotides (Invitrogen, catalog number HSS102781: 5′-AACACCUGGAAGCAUAAUCUUGGCC-3′, HSS102782: 5′-UAAACAACCAGUCACCUUGGGAUCC-3′, HSS102785: 5′-GAAGUCACAACAGAGUGCAGGCAAA-3′). siRNA were transfected using Lipofectamine RNAiMAX (Invitrogen) in Opti-MEM medium (Invitrogen) according to the manufacturer’s recommended procedures.

For stable DKC1 KD, short hairpin oligonucleotides were inserted into a modified version of the pSuper.puro expression vector in which the puromycin resistance gene was substituted with blasticidin resistance gene—pSuper.blast—(a kind gift of Dr Kenneth B. Marcu), according to the manufacturer’s instructions (OligoEngine, Seattle, WA). The sequence of the short hairpin oligonucleotide targeting DKC1 mRNA was 5′-ccaaggtgactggttgtttaat-3′. As a negative control, an empty pSuper.blast vector was used. Retroviruses were produced by transient transfection of pSuper.blast retrovectors into Phoenix A packaging cells (kindly provided by Dr Gary Nolan) and used to infect MCF7. Stable retroviral-transduced populations of cells were selected in growth media supplemented with blasticidin (Invitrogen).

### RNA extraction and real-time polymerase chain reaction

Total RNA was extracted from MCF7 and MDA-MB231 cells 96 h after siRNA transfection using a TRI reagent (Ambion) according to the manufacturer’s instructions. RNA was reverse-transcribed using the High-Capacity cDNA Archive Kit (Applied Biosystems) following the manufacturer’s instructions. Real-time polymerase chain reaction (PCR) analysis was performed in a Gene Amp 7000 Sequence Detection System (Applied Biosystems) using the TaqMan approach. For each sample, three replicates were analyzed. Sets of primers and fluorogenic probes specific for DKC1 (catalog number Hs00154737_m1), VEGF (Hs00900054_m1) and ®-Actin (Hs99999903_m1) mRNAs were purchased from Applied Biosystems. The relative amounts of the studied target genes were calculated using the expression of human β-glucuronidase (for total mRNA analysis, Applied Biosystems—4326320E) and 18S RNA (for polysomal analysis—Hs99999901_s1) as endogenous controls. The final results were determined by the 2^-ΔΔCt method.

### mRNA transfection

Capped mRNA was transcribed from linearized pR-VEGF-IRES-F (gift of Prof. G.J. Goodall) (18), pR-CrPV-IRES-F (gift of Dr Ruggero) (9), pF-EMCV-IRES-R (gift of Prof. A.C. Palmenberg) (19), pR-HCV-IRES-F (gift of Prof R.E. Lloyd) (20), pRHSP70-IRES-F (gift of Prof. I.N. Shatsky) (21), pR-c-MYC-IRES-F (22) and pR-p53-IRES-F plasmids (gift from Dr Mazumder) (23), using the mMessage mMachine T7, SP6 or T3 kits (Ambion). Cells were transfected with 0.4 μg RNA/sample using Lipofectamine 2000 (Invitrogen) following the manufacturer’s instructions. After an 8-h transfection, cells were harvested and analyzed with a dual-luciferase assay kit (Promega) following the manufacturer’s instructions.

### Western blot

Whole cell protein extracts and subsequent sodium dodecyl sulphate-polyacrylamide gel electrophoresis and immunoblot analysis were carried out according to standard procedures. The following antibodies were used: anti-dyskerin (Santa Cruz Biotechnology) anti-β-actin (Sigma-Aldrich), anti-VEGF (Santa Cruz Biotechnology), anti-eIF4G (Cell signaling), anti-Pak2 (Abcam), anti-PTBP1 (Abcam) and anti-SSB/La (Santa Cruz Biotechnology).

### VEGF quantitative evaluation

The quantitative evaluation of VEGF was made by using an enzyme-linked immunosorbent assay (ELISA, Quantikine Kit, R&D) following the manufacturer’s instructions. Results were processed as follows: the average of the duplicate readings for each standard, control and sample were obtained, and the average zero standard optical density was subtracted from that. A standard curve was created by plotting the mean absorbance for each standard on the y-axis against the concentration on the x-axis; a best-fit curve was drawn through the dots on the graph. Data were linearized by plotting the log of VEGF concentrations versus the log of the O.D., while the best-fit line was determined by regression analysis. Sample concentration was determined on the standard curve. If samples were diluted, the concentration read from the standard curve was multiplied by the dilution factor. Results are presented after relative normalization with respect to controls.

### Analysis of protein synthesis in cells

Protein synthesis was measured as the rate of incorporation of labeled leucine during a 30-min incubation of the subconfluent MCF7 and MDA-MB231 cells in a complete medium containing 50 mg/l leucine and trace amounts of [3H]leucine.

The final level of protein synthesis was obtained after normalizing the radioactivity to the total amount of proteins rescued.

### Isolation of polyribosomal mRNA

Subconfluent cells were harvested and pelleted. The cellular pellet was lysed in 2 volumes of 10 mM Tris–HCl, pH 7.4, 10 mM NaCl, 3 mM MgCl_2_, 0.5% NP-40 for 10 min at 4°C. Then lysates were centrifuged at 14 000*g* for 10 min at 4°C. The supernatant was stratified onto a 15–50% sucrose gradient in 30 mM Hepes/KOH (pH 7.5), 80 mM KCl, 1.8 mM Mg-Acetate and centrifuged at 4°C for 15 h at 40 000*g*. From the gradients, 1-ml fractions were collected, reading their 260 nm absorbance. Polyribosomal and prepolysomal fractions were pooled and centrifuged at 10 000*g* for 15 h at 4°C. RNA was extracted from pellets using the TRI reagent.

### Clonogenic assay

MCF7 ctrl and shDKC1 cells were seeded in duplicate in a six-well plate at the concentration of 250 cells/well in normal medium. The day after, cells were treated with 100 nM PTC299 and maintained in such condition, replacing medium and 100 nM PTC299 every 2 days. After 7 days, cells were fixed in methanol and stained with 0.5% crystal violet in 25% methanol for 20 min. Finally, the colonies were counted. Results are presented as average of the number of colonies counted in each well for each condition.

### 48S preinitiation complex formation analysis

Analysis of the 48S preinitiation complex formation was performed as described (11,24). Briefly, VEGF-IRES plasmids was linearized, *in vitro* transcribed and radiolabeled using a MAXIscript SP6 kit (Ambion) in the presence of 50 μCi of [α-32P]UTP (Perkin-Elmer). Radiolabeled [^32^P] RNA was incubated with cytoplasmic extracts prepared from DKC1 KD and control MCF7 cells. Cells were harvested and lysed in 10 mM Tris–HCl (pH 8.0), 140 mM NaCl, 1.5 mM MgCl_2_, 20 units of RNasin, 0.25% NP40, 150 μg/ml cycloheximide and 20 mmol/L dithiothreitol (DTT). Protein extracts (50 µl) were preincubated in 100 µl for 2 min at 30°C with the components necessary for translation initiation 1 mM ATP, 10 mM creatine-phosphate, 1 mg/ml creatine phosphokinase, 0.02 mM l-methionine and 12.5 mM HEPES-KOH (pH 7.5) as well as the GTP analogue, β, γ-imido-GTP (GMP-PNP) 4 mM (Sigma), 0.6 mM of cyclohexymide and 0.25 mM of spermidine. Translation initiation complexes (48S) were formed by incubating the mixtures with the 2 × 10^6^ cpm [^32^P]p27 IRES mRNA probe for 15 min at 30°C. Following incubation, the samples were run over 30 ml 10–30% sucrose density gradients on a SW28 Beckman rotor at 20 000 rpm at 4°C for 15 h. Gradients were then fractionated into 750 μl fractions; each was immersed into scintillation fluid and counted on a Wallace scintillation counter. Counts per minute were plotted against the fraction number. Fractions corresponding to preinitiation complex were identified reading the 260 nm O.D. profile of samples similarly prepared except for the addition of the radiolabeled mRNA probe and checking the presence of 18S RNA in the identified peaks.

### Statistical analysis

Mann–Whitney U test or paired Student’s *t*-test, when appropriate, were used for the comparisons among groups. Values for *P* < 0.05 were regarded as statistically significant.

## RESULTS

### Dyskerin KD increases VEGF-IRES–mediated translation

**Figure 1.**
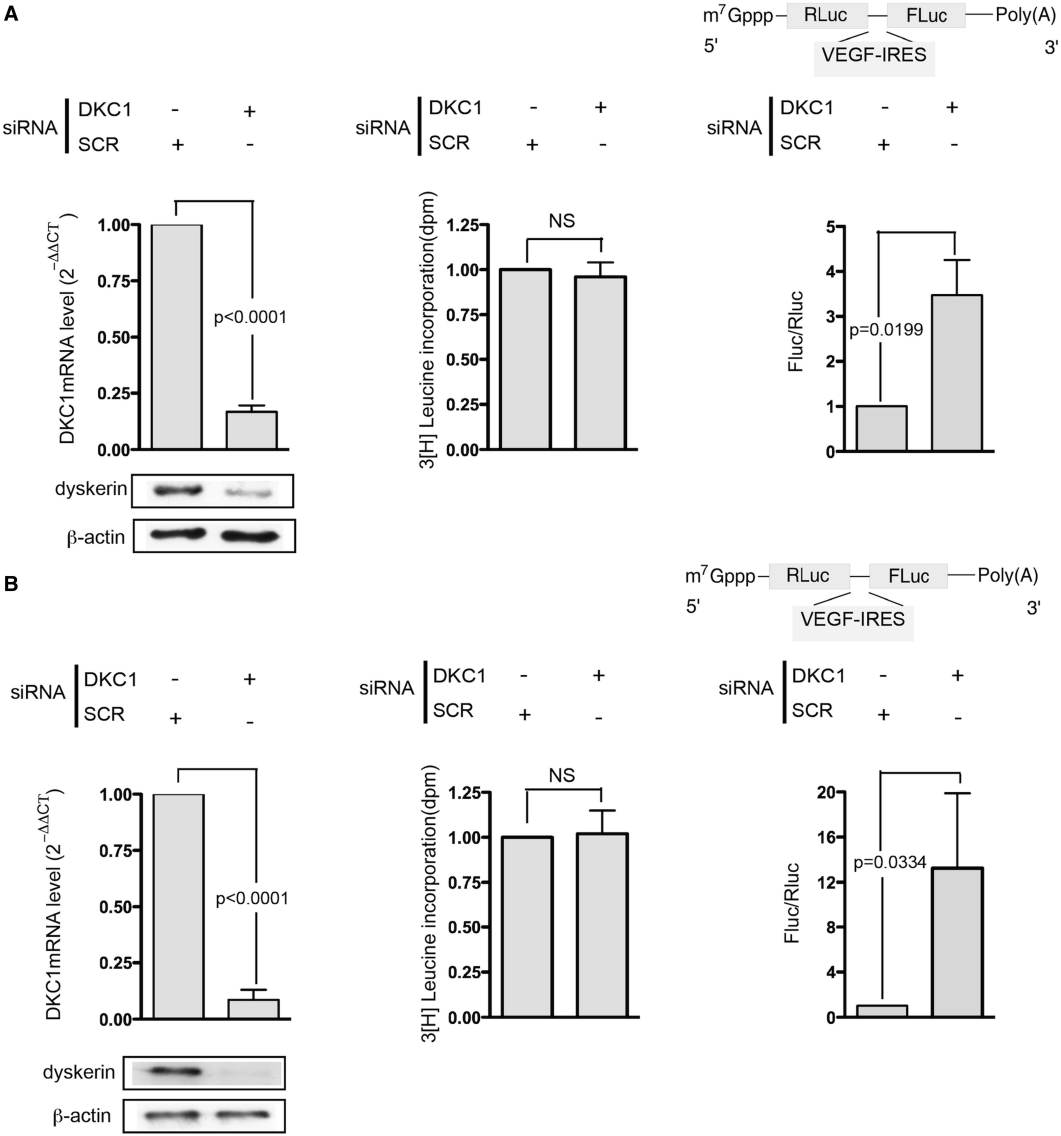
Dyskerin KD stimulates VEGF-IRES–mediated translation. Transient transfection of DKC1-specific siRNA strongly reduced DKC1 mRNA and protein level in MCF7 and MDA-MB231 cells (**A**, left and **B**, left, respectively). [3H]-leucine incorporation indicates that the total protein synthesis is not compromised (A and B, center) after DKC1 KD. IRES-mediated translation was assessed by measuring the FLuc and RLuc activity in MCF-7 and MDA-MB231 (A, right, and B, right, respectively) cells 8 h after the transfection with the bicistronic mRNA transcribed from pRL-VEGF-IRES. siRNA transfection was performed 96 h before cell harvesting. Histograms represent means and SDs from at least three independent experiments. *P* < 0.05 are considered significant. NS = not significant.

Firstly, we transiently reduced DKC1 mRNA levels by specific siRNA transfection in MCF7 and MDA-MB 231 breast cancer cell lines. Ninety-six hours after transfection, DKC1 mRNA decreased to at least 80% of controls and protein levels also reduced (Figure 1A and B, left). In these conditions, the total protein synthesis was not compromised (Figure 1A and B, center). We then evaluated whether the translation mediated by an IRES element described in VEGF mRNA can be influenced by dyskerin levels. In particular, cells were transfected with an *in vitro* transcribed bicistronic mRNA in which the IRES sequence was inserted between the two reporter cistrons (Figure 1A and B, right). The reduction in DKC1 levels significantly increased the VEGF-IRES–mediated translation in both MCF7 and MDA-MB 231 cells (Figure 1A and B, right panels and Supplementary Figure S1). The increase in VEGF-IRES–mediated translation due to dyskerin depletion was also observed under 1% oxygen hypoxia, a condition known to positively regulate VEGF-IRES–mediated translation increases (25) (Supplementary Figure S2) and in primary mouse embryonic fibroblast (Supplementary Figure S3).

### Dyskerin KD drives VEGF mRNA translation

**Figure 2.**
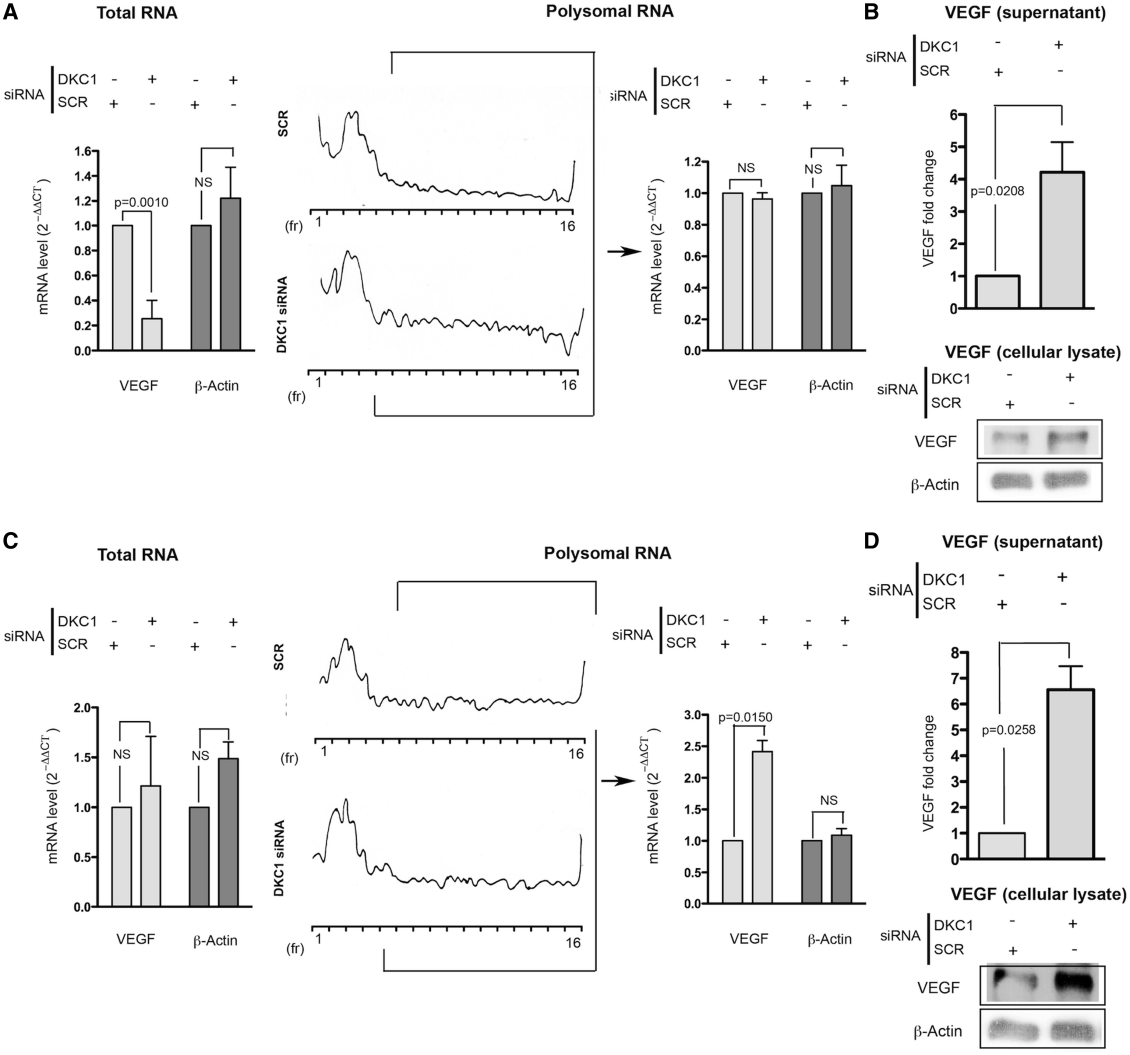
Dyskerin KD drives VEGF mRNA translation in breast cancer cells. Total (left) and polysome-associated (right) VEGF mRNA levels assessed by real-time PCR after DKC1 KD in MCF7 (**A**) and MDA-MB231 cells (**C**). Representative polysomal profiles are shown. VEGF protein levels in supernatant and in whole cell extracts are also reported for MCF-7 (**B**) and MDA-MB231 cells (**D**). siRNA transfection was performed 96 h before cell harvesting. Histograms represent means and SDs from three independent experiments. *P* < 0.05 is considered significant.

To clearly address the effect shown to a specific translational control mechanism, we investigated the levels of VEGF mRNA in DKC1 KD MCF7 and MDA-MB231 and in control cells, in both the total and the polysomal mRNA extracts. Polysomes are clusters of ribosomes associated with mRNAs, indicating that the mRNA is translationally active. It is possible to isolate the polysomal component as far as the 40S, 60S and 80S components on the basis of their sedimentation on a sucrose gradient by ultracentrifugation, as well as to extract the mRNA associated with the different fractions. DKC1 KD significantly decreased the total VEGF mRNA level in the MCF7 (Figure 2A, left) and had no significant effects on total VEGF mRNA levels in the MDA-MB231 (Figure 2C, left). The analysis of the total β-Actin mRNA, an mRNA known not to change on DKC1 KD (10), was included in Figure 2A and C as an appropriate control.

In MCF7 cells, after DKC1 KD, a strong reduction of global levels of VEGF mRNA was observed. This global reduction was not followed by a decrease in the VEGF mRNA recruitment to polysomes. These results are indicative of the occurrence of a relative increase in the recruitment of the VEGF mRNA to polysomal fractions in MCF7 cells after DKC1 KD. (Figure 2A, right). This was also confirmed by the observation that in these cells the reduction of VEGF mRNA levels induced by DKC1 KD specifically reduced the amount of VEGF mRNA in the non-polysomal fractions (Supplementary Figure S4).

In MDA-MB231, DKC1 KD significantly increased the association of the VEGF mRNA to polysomal fractions (Figure 2C, right).

We then investigated whether the observed translational increase of VEGF mRNA might affect VEGF protein levels. By using an ELISA array, we measured the level of the VEGF protein secreted by MCF7 (Figure 2B, top) and MDA-MB231 cells (Figure 2D, top). In addition, to exclude the possibility that DKC1 knock affects the secretion of VEGF more than its synthesis, we performed a western blot analysis on cell lysates from both MCF7 and MDA-MB231 cells (Figure 2B and D bottom, respectively). The obtained results indicated that DKC1 KD led to an increase in the secreted and cellular amount of VEGF protein in both cell lines.

### The increased VEGF secretion in DKC1 KD cells is due to the upregulation of VEGF mRNA IRES-mediated translation

**Figure 3.**
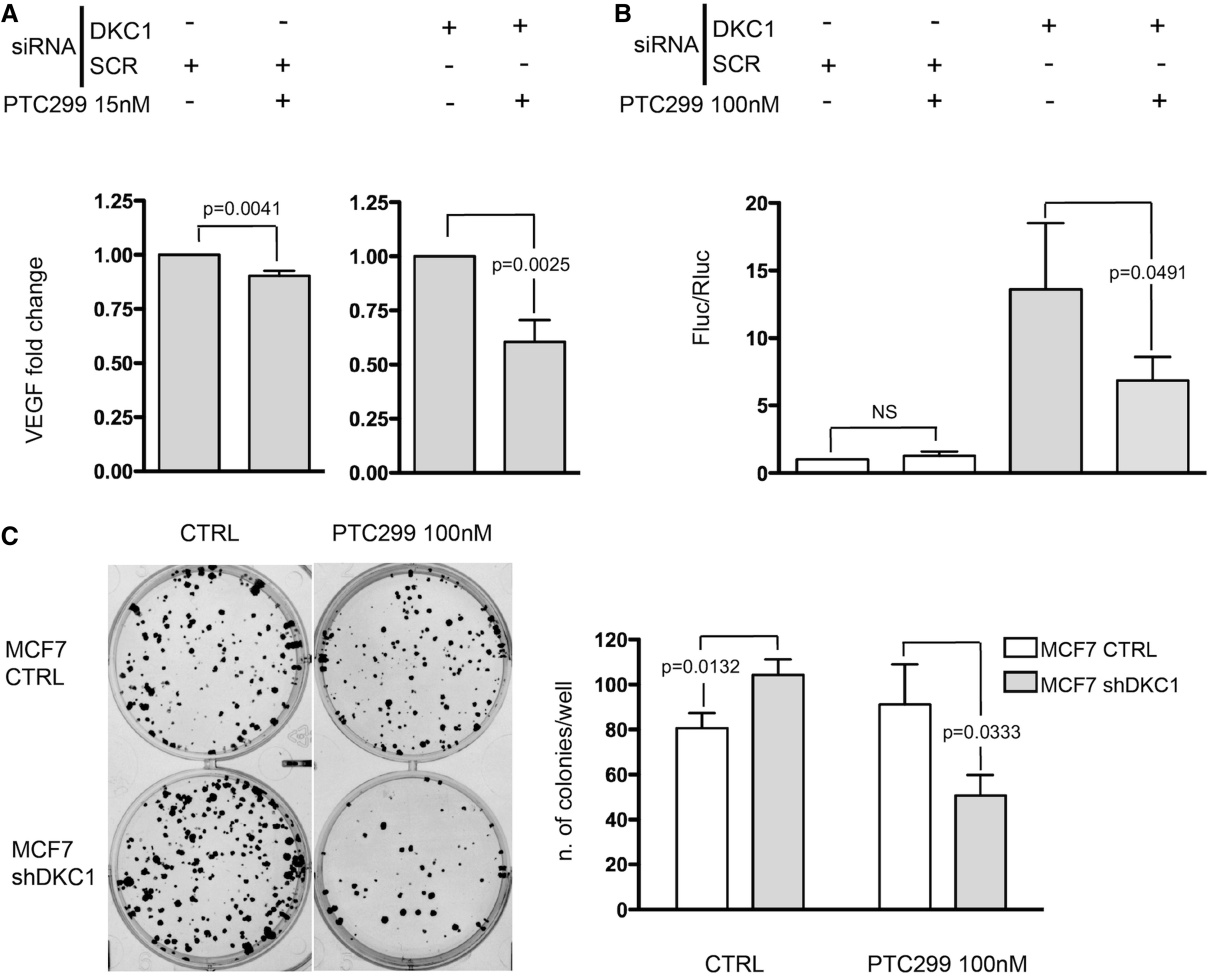
The increased VEGF secretion in DKC1 KD cells is due to the up-regulation of VEGF mRNA IRES-mediated translation. (**A**) Histograms show the level of VEGF protein detected using an ELISA array. Experiments were performed in MCF-7 control (left) and DKC1 KD (right) cells treated with 15 nM PTC299 for 72 h. VEGF secretion of PTC299 treated cells is normalized on the correspondent untreated cells; for the effect of DKC1 KD on VEGF secretion on MCF7 cells see Figure 2A (**B**) VEGF-IRES–mediated translation measured in MCF7 control and DKC1 KD cells after 72 h of 100 nM PTC299 treatment. siRNA transfection was performed 96 h before cell harvesting. (**C**) Representative image (left) and a summarizing graph (right) from clonogenic assays performed with control (empty vector) and shDKC1 MCF7 cells. Histograms represent means and SDs from three independent experiments. *P* < 0.05 is considered significant. NS = not significant.

To determine the reason for the increase in VEGF secretion, we treated MCF7 DKC1 KD and control cells with PTC299, a small molecule that posttranscriptionally impairs the VEGF synthesis (Dr T. Davis, personal communication). PTC299 has been proposed in cancer therapy to target the 5′UTR of the VEGF mRNA by downregulating the protein level (26). Cells were treated with 15 nM PTC299 for 72 h after DKC1 KD and the VEGF protein levels were measured. Obtained results indicated that PTC299 treatment only slightly reduced the VEGF secretion in control cells, while the treatment was much more efficient on MCF7 DKC1 KD cells (Figure 3A), when the VEGF-IRES–mediated translation is upregulated. Accordingly, treatment with PTC299 100 nM for 72 h had no clear effect in downregulating VEGF mRNA IRES-mediated translation in control cells. On the other side, in MCF7 DKC1 KD cells, in which an upregulation of VEGF mRNA translation takes place, VEGF-IRES–mediated translation is significantly impaired (Figure 3B). The values of the activity of each luciferase are shown in Supplementary Figure S5. In addition, to provide functional insights into the relevance of increased VEGF-IRES–mediated translation on DKC1 KD in breast cancer cells, we assessed how the clonogenic potential of breast cancer cell lines is modified by DKC1 KD. For this purpose, we generated a construct to mediate the stable DKC1 KD. In MCF7 cells, the transduction of the construct induced ∼50% reduction of dyskerin levels (Supplementary Figure S6). Our results indicate that the stable DKC1 KD significantly increased the clonogenic potential of MCF7 cells. The observation that the increase in colony formation is reverted on treatment with PTC299 (Figure 3C) supports, at least in part, its ascription to the effect of DKC1 KD on VEGF synthesis.

### DKC1 KD increases VEGF-IRES recruitment to 48S preinitiation complex

**Figure 4.**
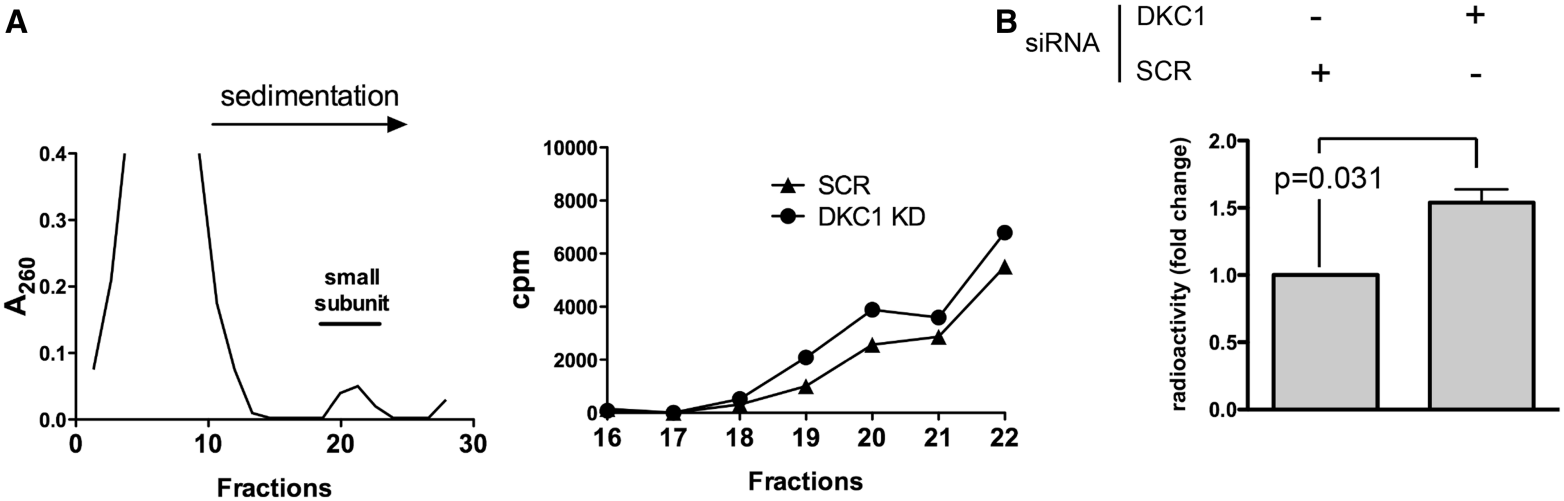
DKC1 KD increases VEGF-IRES recruitment to 48S preinitiation complex. (**A**) Left: representative profile at 260 nM O.D. obtained from MCF7 cytoplasmic extracts: fractions 18–21 were considered to correspond to the small ribosomal subunits. Right: Representative profile of a sucrose density gradient reporting the radioactive intensity per fraction in from DKC1 KD (circles) and control (SCR—triangles) cells extracts, respectively. Peaks of radioactivity coinciding with the identified fractions containing the 48S complexes was generated when MCF7 cytoplasmic extracts were incubated with a [^32^P]VEGF IRES mRNA probe. (**B**) Histogram represent mean and SD of the radioactivity measured in the identified peaks for DKC1 KD and control (SCR) cells extracts. siRNA transfection was performed 96 h before cell harvesting. *P* < 0.05 is considered significant.

To try to explain the mechanism underlying the upregulation of VEGF-IRES–mediated translation in cells after DKC1 KD, we reconstituted the formation of the 48S mRNA initiation complex on the VEGF IRES using cytoplasmic extracts from DKC1 KD and control MCF7 cells. This allowed to investigate whether DKC1 depleted cells, which are known to be impaired in the formation of the complex on the IRES element of p27 (11) and CrPV RNAs (27), have instead an advantage in this critical event of the initiation process when challenged with the VEGF IRES element. We found that the total amount of VEGF IRES-containing preinitiation complexes formed by lysates from in DKC1 KD cells is significantly increased as compared with control cells (Figure 4). Importantly, a control experiment performed with the same lysates showed that the formation of the preinitiation complex mediated by CrPV IRES is impaired as expected (Supplementary Figure S7). To provide an explanation for these results, we evaluated if the expression of factors that could affect VEGF-IRES–mediated translation is modified by dyskerin depletion. In particular, we evaluated the expression of a limited list of known IRES trans acting factors known to potentially favor VEGF IRES translation including PAK2, PTBP1 and SSB/La Autoantigen (28,29). In addition, we also evaluated the expression of eIF4GI, a canonical initiation factor known to play a role in VEGF-IRES–specific initiation (30). DKC1 KD did not induce the expression of any of these proteins (see supplementary Figure S8), therefore indicating that the surge in VEGF-IRES–mediated translation initiation could not be ascribed to their upregulation.

### The reduction of dyskerin levels differentially affects IRES-mediated translation of viral and cellular IRESs

**Figure 5.**
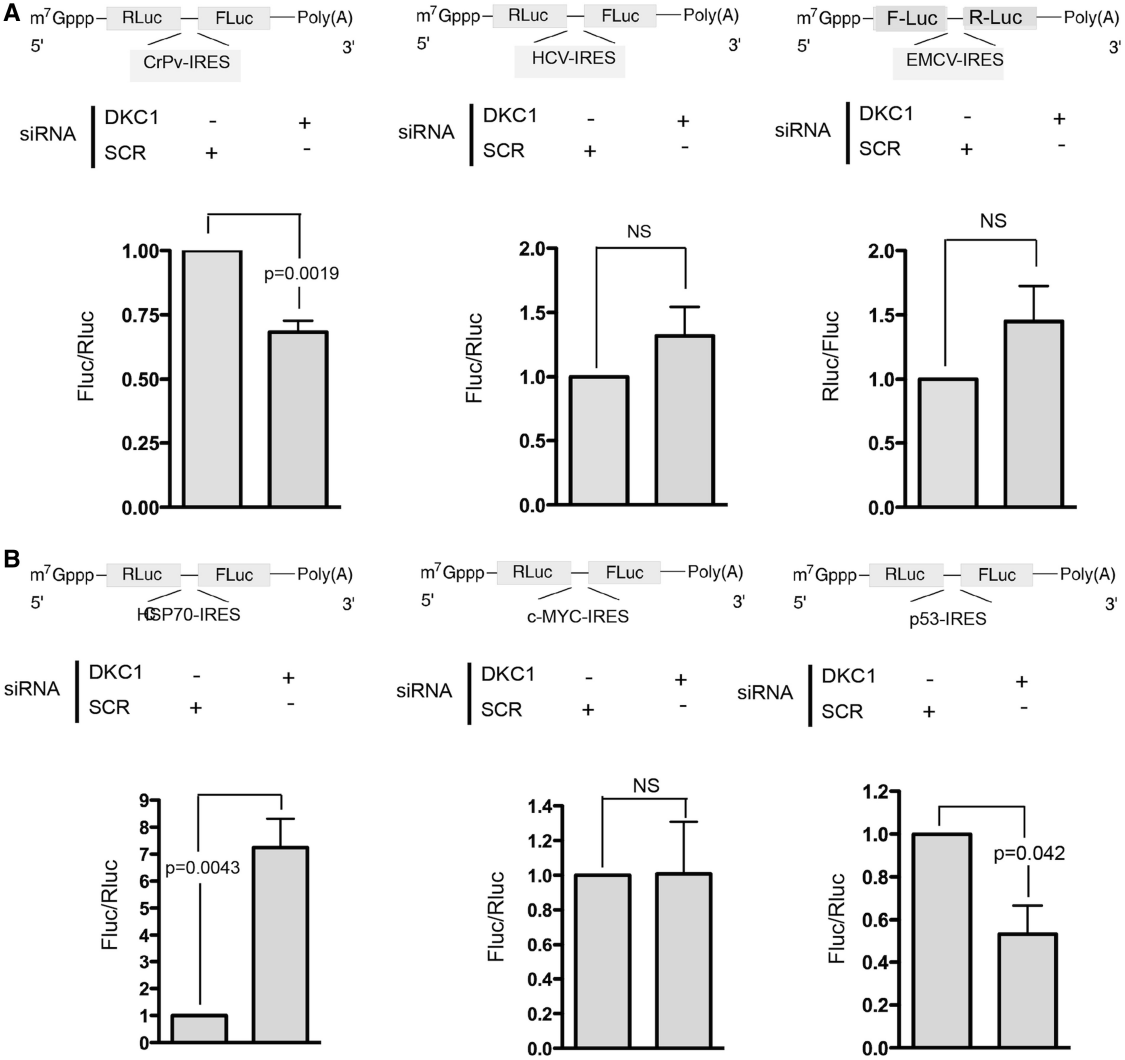
Reduction of dyskerin levels differentially affects IRES-mediated translation of viral and cellular IRESs. (**A**) IRES-mediated translation assessed by measuring the FLuc and RLuc activity in MCF-7 DKC1 KD cells, 8 h after transfection with a bicistronic mRNA transcribed from viral pR-CrPV-IRES-F (left), pR-HCV-IRES-F (center) and pF-EMCV-IRES-R (right). (**B**) IRES-mediated translation assessed by measuring the FLuc and RLuc activity in MCF-7 DKC1 KD cells 8 h after transfection with a bicistronic mRNA transcribed from cellular pR-HSP70-IRES-F (left), pR-c-MYC-IRES-F (center) and pR-p53IRES-F (right). siRNA transfection was performed 96 h before cell harvesting. Histograms represent means and SDs from three independent experiments. *P* < 0.05 is considered significant. NS = not significant.

In contrast to what has been reported so far, the present results demonstrate for the first time that the reduction of dyskerin levels can upregulate the IRES translation initiation of specific cellular mRNAs. To have a more general picture of the alteration of IRES-mediated translation induced by DKC1 KD, we investigated its effect on the translation of a panel of viral IRESs. We studied the translation of viral IRESs representative of different known viral IRES types. In particular, we used the encephalomyocarditis virus (EMCV) IRES for Type 2, the hepatitis C virus (HCV) IRES for Type 3 and the Cricket paralysis virus (CrPV) IRES for Type 4. Of note, type 1 IRESes, although they differ in their RNA structure and in their way to recruit the ribosome, share a number of similarities with those of type 2 (31). The reduction of dyskerin levels in MCF-7 cells led to a significant decrease in the translation mediated by the CrPV IRES (Figure 5A, left), as already described. In contrast, DKC1 KD did not significantly change the translation mediated by the HCV (Figure 4A, center) and EMCV IRESs (Figure 5A, right). To demonstrate that the translation initiation of cellular IRESs can have a similar heterogeneous behavior after DKC1 KD, we analyzed the translation of IRES elements present in mRNA encoding proteins that can play important roles supporting the growth of breast cancer cells, such as those present in the oncogene c-myc and the heat shock protein 70 (HSP70) mRNAs. DKC1 KD strongly increased the IRES-mediated translation of the HSP70 (Figure 5B, left), did not affect the translation initiation mediated by the c-myc-IRES (Figure 5B, center). In the same experimental conditions, DKC1 KD reduced p53-IRES–mediated translation, as already reported (Figure 5B, right) (10). These results confirm that dyskerin depletion can differentially control the translation mediated by different cellular IRES elements. The values of the activity of each luciferase are shown in Supplementary Figure S9.

## DISCUSSION

Our results indicate that the lack of dyskerin can activate the translation of selected IRES-containing mRNAs. In particular, we demonstrated that dyskerin depletion stimulates the IRES-mediated translation of the mRNA encoding for the VEGF. In addition, dyskerin depletion results in the differential regulation of the translation mediated by IRES sequences present in different viral mRNA types as well as in specific cellular mRNAs.

It has been previously demonstrated that dyskerin clearly has a role in controlling the IRES-mediated translation initiation (8). In particular, all the data published support the idea that dyskerin depletion downregulates the IRES-mediated translation (9–11). This happens to the mRNAs encoding the anti-apoptotic factors Bcl-xL and XIAP (9) and the tumor suppressors p27 (8,11) and p53 (10,32). Here we demonstrated for the first time that on different mRNAs types, dyskerin KD can upregulate the translation of selected mRNAs containing IRES elements. DKC1 KD significantly increased the ratio between the activities of two reporters of a bicistronic construct containing the VEGF-IRES in both MCF7 and MDA-MB231 breast cancer cells. The observed increase in VEGF-IRES translation is different in the two cell lines being much more evident in MDA-MB231 cells. This difference may reflect the differential capability of the two cell lines to grow in low-oxygen conditions and/or to synthesize VEGF (33). The observed increase of the reported ratio is due mainly to an increase of VEGF-IRES–dependent reporter activity after DKC1KD; however, in some experiments also a slight downregulation of cap-dependent reporter activity could be detected. For the intrinsic characteristics of the controlled assay also this element affects the obtained ratio. However, the analysis of the individual reporter activities indicates that the ratio increase cannot be considered simply as the consequence of an overall decrease in cap-dependent translation. This is also corroborated by the data obtained from the analysis of polysomal fractions: while DKC1 KD significantly decreased the total VEGF mRNA level in the MCF7, the relative recruitment of VEGF mRNA to polysomal fraction was increased. In the MDA-MB231, the results are even more evident, as there are no significant differences in the rate of the total VEGF mRNA level, whereas an important increase in the amount of VEGF mRNA recruited to polysomes is observed. The increase in VEGF mRNA translation is associated with an increased VEGF protein secretion by both DKC1 KD MCF7 and MDA-MB231 cells. In addition, the increase in VEGF secretion is clearly caused by the VEGF mRNA IRES-mediated translation as it is reverted after treatment with the small molecule PTC299. Indeed, in contrast with the common agents that block the distal portion of the VEGF signaling pathway (17), PTC299 is a drug that block the production of tumor VEGF at the posttranscriptional level [Dr T. Davis, personal communication and (26)]. Additionally, by means of a stable shRNA approach, we observed that the reduction of dyskerin levels induces an increase of the clonogenic potential of breast cancer cells. This more aggressive phenotype appears to be, at least to some degree, dependent on the increase of VEGF secretion, as it is reverted on PTC299 treatment.

The analysis of 48S preinitiation complex formation further confirmed the increase in VEGF IRES translation in DKC1-depleted cells with an independent assay and provided functional insights of the mechanism that may give advantage for VEGF-IRES–mediated translation. Indeed, the formation of the complex, a critical step of mRNA translation initiation, is favored on the VEGF IRES element. As for the other assays included in this study, VEGF IRES translation in cells lacking dyskerin is different to what is reported for other IRES elements (e.g. p27 IRES; CrPV IRES) (11,27). However, as the formation of the preinitiation complex constitutes only one of the steps of mRNA translation, it cannot be excluded that dyskerin KD could act also at other levels.

Given the known role of dyskerin in rRNA pseudouridylation, the differences shown in the regulation of IRES-mediated translation may be due to the qualitative changes in the ribosomes caused by DKC1 KD. Even if an intrinsic functional ribosomal defect has never been clearly demonstrated, the analysis of the mRNA associated with the polysomal fractions indicates that the changes in protein expression may be ascribed to the translational activity. To explain the observed results, a possible mechanism that may be hypothesized is that the defect in the pseudouridylation may lead to ribosome structural modifications, which in turn affect mRNA translation (27,34). On the other hand, it has recently been reported that a cytoplasmic isoform of dyskerin can be expressed in human cells (35). The function of such cytoplasmic isoform has not been fully elucidated, and its possible involvement in mRNA translation regulation cannot be excluded. On this regard, as the siRNA used in our study can target both the nuclear and the cytoplasmic dyskerin isoforms, it is not possible to exclude that the changes in VEGF mRNA translation are due to the KD of the cytoplasmic isoform.

In addition, the siRNA used in the study could have also induced some of the observed effects through an unspecific targeting of other transcripts. However, an analysis of the effect of DKC1 KD performed with different siRNA oligo separately on the expression of a list of target genes considered in this study excluded this possibility (see Supplementary Figure S10).

It is also important to mention that some reports suggest the idea of dyskerin as a tumor suppressor (8–11). This viewpoint is supported by the data presented here; also, in fact, the defect in dyskerin function can modulate the IRES-mediated translation of different subgroups of cellular mRNAs while simultaneously driving both a reduction in the expression of factors limiting cell proliferation, such as tumor suppressors, and an increase in those promoting cancer cell growth, such as growth factors.

Lastly, our results consolidated the role of dyskerin in the control IRES-mediated translation initiation. The mechanism of IRES-dependent translation activation remains little understood. On the basis of the structures and the involvement of the canonical initiation factors, the best-studied viral IRESs are divided into four types: Type 1 (e.g. poliovirus) and Type 2 (e.g. EMCV) IRESs require eIF4G and eIF4A but do not require eIF4E for their activity; Type 3 (e.g. HCV) IRESs require eIF3 but not eIF4F; Type 4 IRESs (CrPV) can support initiation without any canonical initiation factors at all (36). As with the viral counterparts Types 1 and 2, cellular IRES elements (37) have less necessity for initiation factors eIF4E and eIF4G. However, certain cellular IRESs, such as the VEGF, seem to have a strong necessity for eIF4G, while some others are dependent on the initiation factors eIF3 and eIF4A (28). To date, it remains unclear whether cellular IRES-mediated translation may have distinct features with respect to viral IRES-mediated translation and whether cellular IRESs can be classified in a way similar to viral IRESes (38). To investigate potential functional similarities between VEGF-IRES and HSP70-IRES, the two IRES elements that are both positively regulated by DKC1 KD, we investigated if HSP70-IRES translation is affected by the treatment PTC299 similarly to what occurs to VEGF-IRES. The treatment with PTC299 had, however, no significant effect on HSP70-IRES–mediated translation (Supplementary Figure S11), indicating that effect of PTC299 on VEGF-IRES–mediated translation is specific.

Our results show that reduced dyskerin levels induce translational alterations that differentially remodulate IRES-mediated translation; thus, they indicate that the translation initiation mediated by cellular IRESs may involve different mechanisms that can be functionally classified on the basis of the effect of dyskerin downregulation on their activity.

## SUPPLEMENTARY DATA

Supplementary Data are available at NAR Online, including [39].

## FUNDING

Association for International Cancer Research (AICR) [09-0083]; Associazione Italiana per la Ricerca sul Cancro (AIRC) [IG-11416]; Pallotti Legacy for Cancer Research (to L.M.). Funding for open access charge: Pallotti Legacy for Cancer Research.

*Conflict of interest statement.* None declared.

## Supplementary Material

Supplementary Data
