# Supplementary material for: Dyskerin depletion increases VEGF mRNA internal ribosome entry site-mediated translation
Source: Nucleic Acids Res. 2013 Jul 1;41(17):8308–18. doi: 10.1093/nar/gkt587 (PMC3783170; doi:10.1093/nar/gkt587)
Supplement: Supplementary Data [file supp_41_17_8308__index.html]

Dyskerin depletion increases VEGF mRNA internal ribosome entry site-mediated translation — Dyskerin depletion increases VEGF mRNA internal ribosome entry site-mediated translation — Supplementary Data 

# Dyskerin depletion increases VEGF mRNA internal ribosome entry site-mediated translation

## 

files

**Files in this Data Supplement:**

- Supplementary Data - pdf file
