# Supplementary material for: Dyskerin depletion increases VEGF mRNA internal ribosome entry site-mediated translation
Source: Nucleic Acids Res. 2013 Jul 1;41(17):8308–18. doi: 10.1093/nar/gkt587 (PMC3783170; doi:10.1093/nar/gkt587)
Supplement: Supplementary Data [file supp_gkt587_nar-02298-mrna-a-2012-File007.pdf]

Supplementary Figure S1

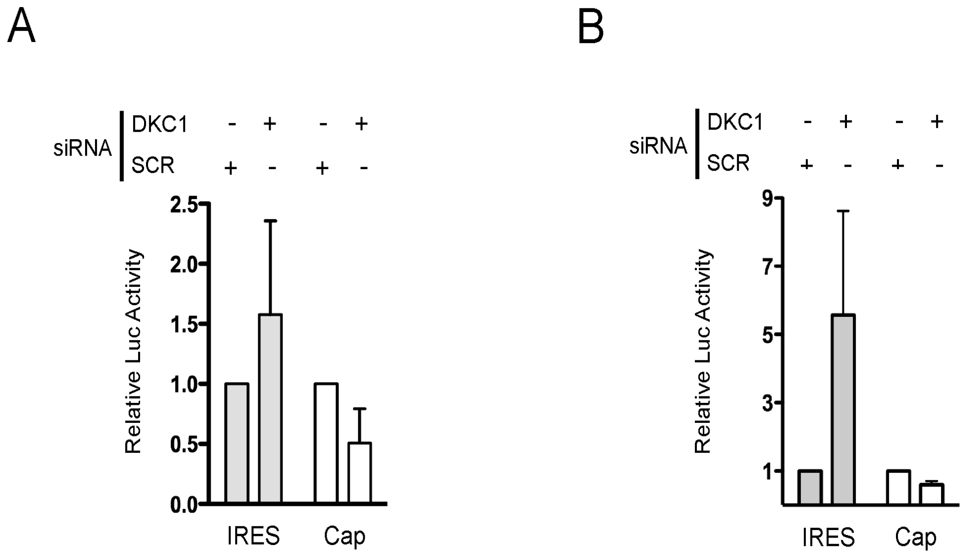

Figure S1. VEGF IRES mediated translation after DKC1 KD. The values obtained for FLuc (IRES-) and RLuc (cap-) activities after transfection with the bicistronic mRNA transcribed from pRL-VEGF-IRES measured in MCF-7(A) and MDA-MB231(B) cells are reported. Values were normalized to those of relevant controls. Cells were harvested 8 hours after the transfection with the bicistronic mRNA. siRNA transfection was performed 96 hours before cell harvesting. Histograms represent means and SDs from at least three independent experiments.

Supplementary Figure S2

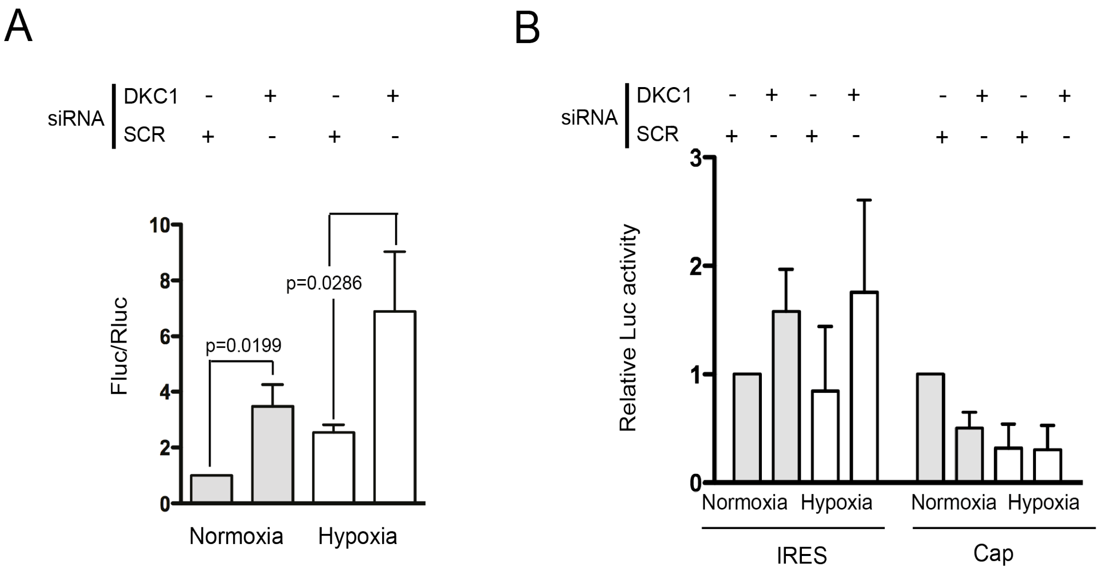

Figure S2. Dyskerin knock-down increases VEGF-IRES mediated translation during hypoxia. IRES-mediated translation was assessed by measuring the FLuc and RLuc activity in MCF-7 cells (A) 8 hours after the transfection with the bicistronic mRNA transcribed from pRL-VEGF-IRES. MCF7 were grown both in normal oxygen concentration and inside a hypoxia chamber at 1% oxygen for 72h. siRNA transfection was performed 96 hours before cell harvesting. Histograms represent means and SDs from three independent experiments. P value <0.05 are considered significant. Values obtained for IRES- (FLuc activity) and cap-(RLuc activity) reporter are shown (B).

Supplementary Figure S3

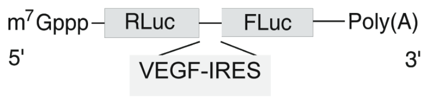

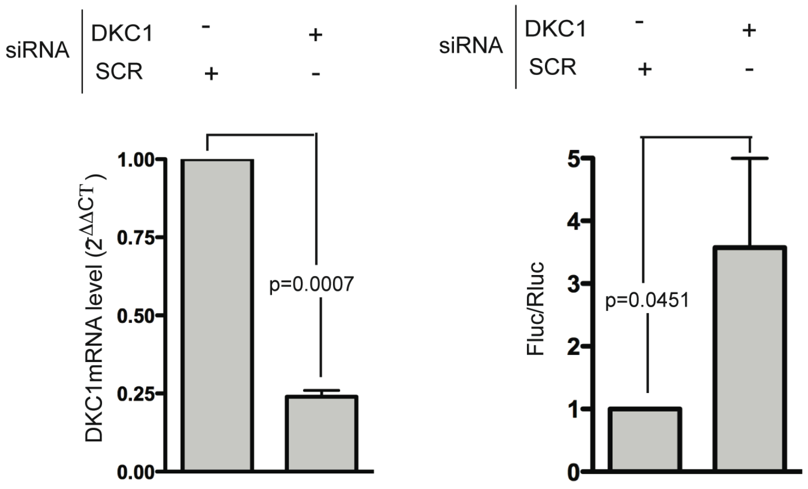

Figure S3. Dyskerin knock-down stimulates VEGF-IRES mediated translation in Mouse Embrionic Fibroblast.

Transient transfection of DKC1 specific siRNA strongly reduced DKC1 mRNA and protein level in MEF (left). IRES-mediated translation was assessed by measuring the FLuc and RLuc activity (right) 8 hours after the transfection with the bicistronic mRNA transcribed from pRL-VEGF-IRES. siRNA transfection was performed 96 hours before cell harvesting. Histograms represent means and SDs from three independent experiments. P value <0.05 are considered significant.

Supplementary Figure S4

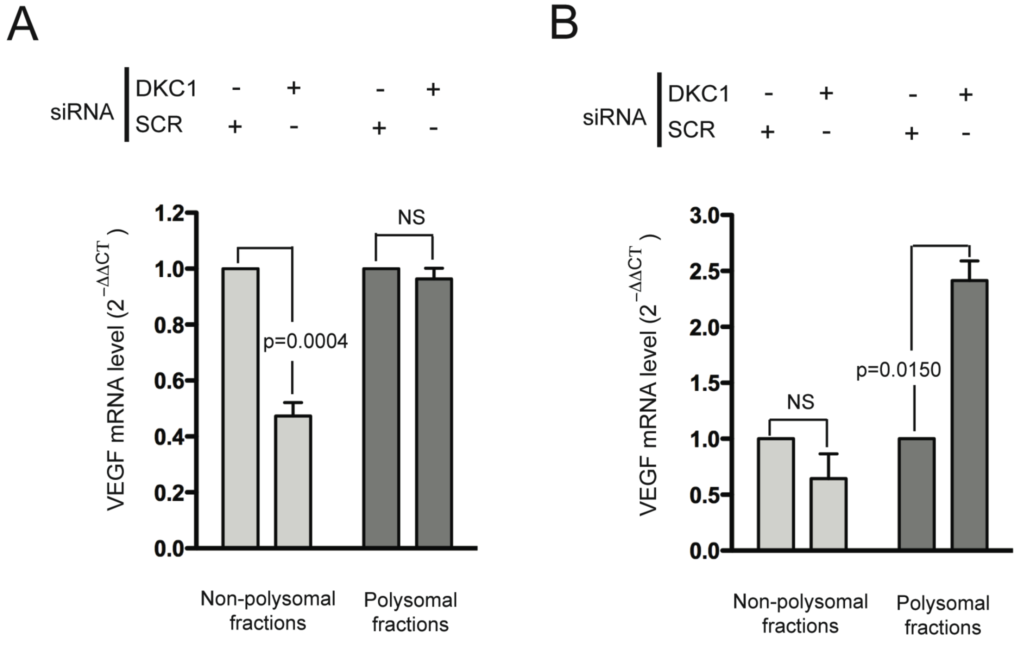

Figure S4. Dyskerin knock-down drives VEGF mRNA translation trough polysomal recruitment. Pre-polysomal and polysomal-associated VEGF mRNA levels assessed by real time PCR after DKC1 KD in MCF7 (A) and MDA-MB231 cells (B). The relative amounts of the studied target gene were calculated using the expression of 18S RNA as endogenous controls. The final results were determined by the 2^-ΔΔCt method. siRNA transfection was performed 96 hours before cell harvesting. Histograms represent means and SDs from three independent experiments. P value <0.05 are considered significant. NS=not significant.

Supplementary Figure S5

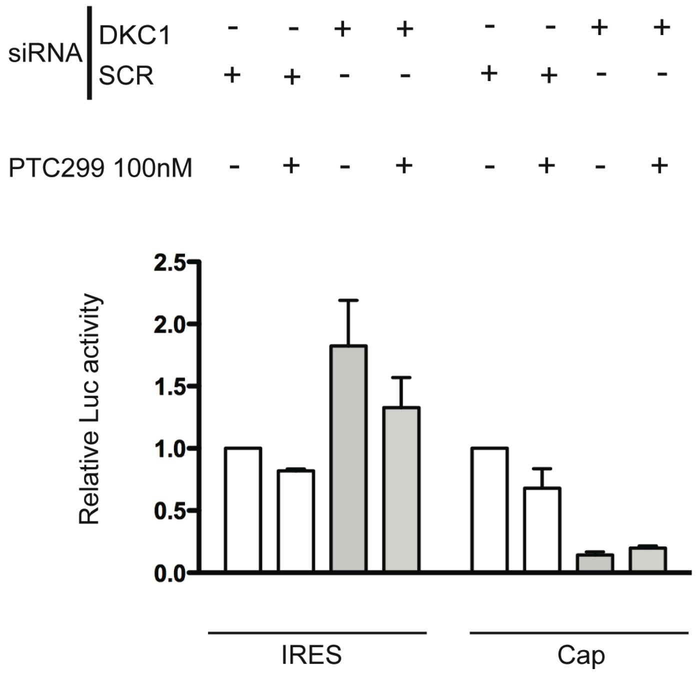

Figure S5. VEGF IRES mediated translation after 100 nM PTC299 treatment. The values obtained for FLuc (IRES-) and RLuc (cap-) activities transfected with the bicistronic mRNA transcribed from pRL-VEGF-IRES in MCF-7 cells treated with 100 nM PTC299 for 72 hours are reported. Values were normalized to those of relevant controls. Cells were harvested 8 hours after the transfection with the bicistronic mRNA transcribed from pRL-VEGF-IRES. siRNA transfection was performed 96 hours before cell harvesting. Histograms represent means and SDs from three independent experiments.

Supplementary Figure S6

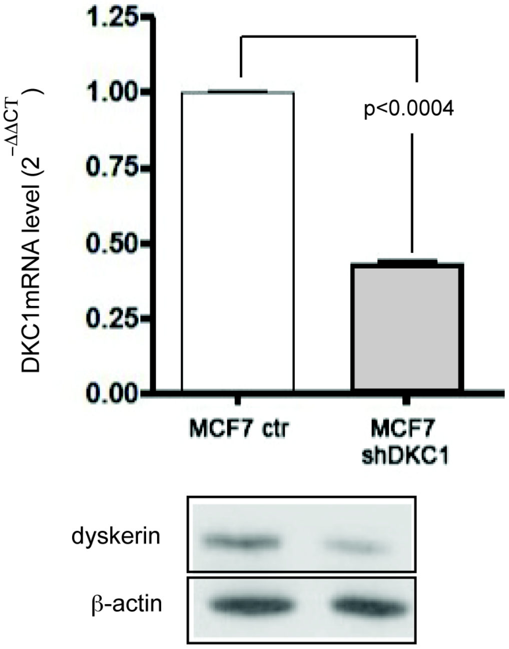

Figure S6. Evaluation of stable DKC1 KD efficiency on MCF7 cells. DKC1 levels evaluated by real-time -PCR (top) and western blot analysis (bottom). Histograms represent means and SDs from three independent experiments. P value <0.05 are considered significant.

Supplementary Figure S7

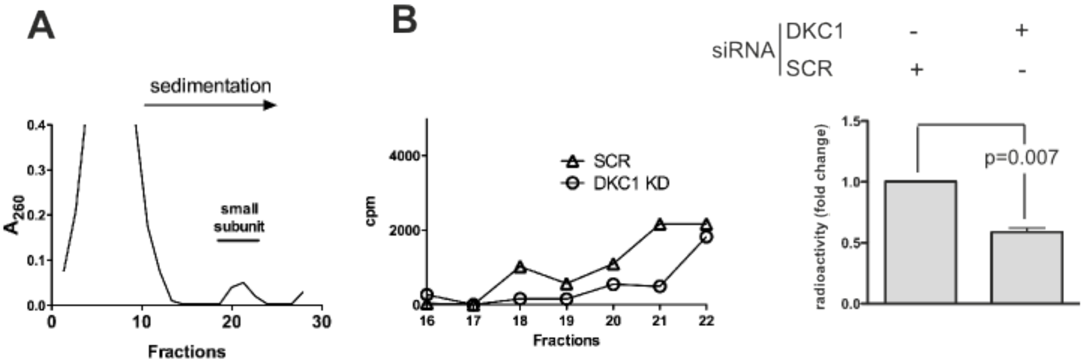

Figure S7. DKC1 KD reduces CrPV-IRES recruitment to 48S preinitiation complex. (A) Left: representative profile at 260 nM O.D. obtained from MCF7 cytoplasmic extracts: fractions 18 to 21 were considered to correspond to the small ribosomal subunits. Right: Representative profile of a sucrose density gradient reporting the radioactive intensity per fraction in from DKC1 KD (circles) and control (SCR - triangles) cells extracts, respectively. Peaks of radioactivity coinciding with the identified fractions containing the 48S complexes was generated when MCF7 cytoplasmic extracts were incubated with a [32P]CrPV IRES mRNA probe. (B) Histogram represent mean and SD of the radioactivity measured in the identified peaks for DKC1 KD and control (SCR) cells extracts. siRNA transfection was performed 96 hours before cell harvesting. P value <0.05 are considered significant.

Supplementary Figure S8

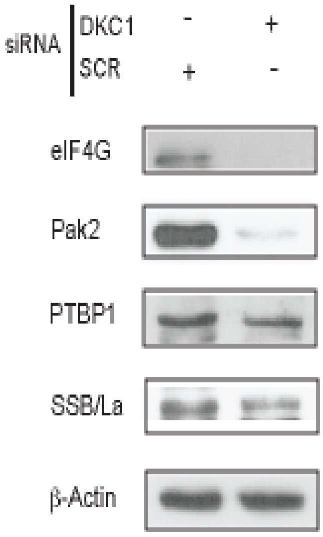

Figure S8 Evaluation of trans-acting factors involved in IRES mediated initiation after DKC1 knock down

Western blot analysis of EIF4G, Pak2, PTBP1, SSB/La proteins in MCF7 cell extracts after dyskerin KD.

These results indicated that DKC1 knock down downregulates the expression of eIF4G and Pak2. Despite this modulation of expression, cap and IRES translation were not generally strongly down-regulated (see Supplementary Figure S1 and S9). The lack of a drastic downregulation of cap dependent translation following DKC1 KD may be explained deeming that eIF4G is generally not considered a limiting component in the formation of the eIF4F initiation complex (39) On the other hand, regarding those IRESes requiring eIF4G and PAK2, the observed effects may explained considering the combined effect of DKC1 KD on ribosomal pseudouridylation and on eIF4G and ITAFs expression.

Supplementary Figure S9

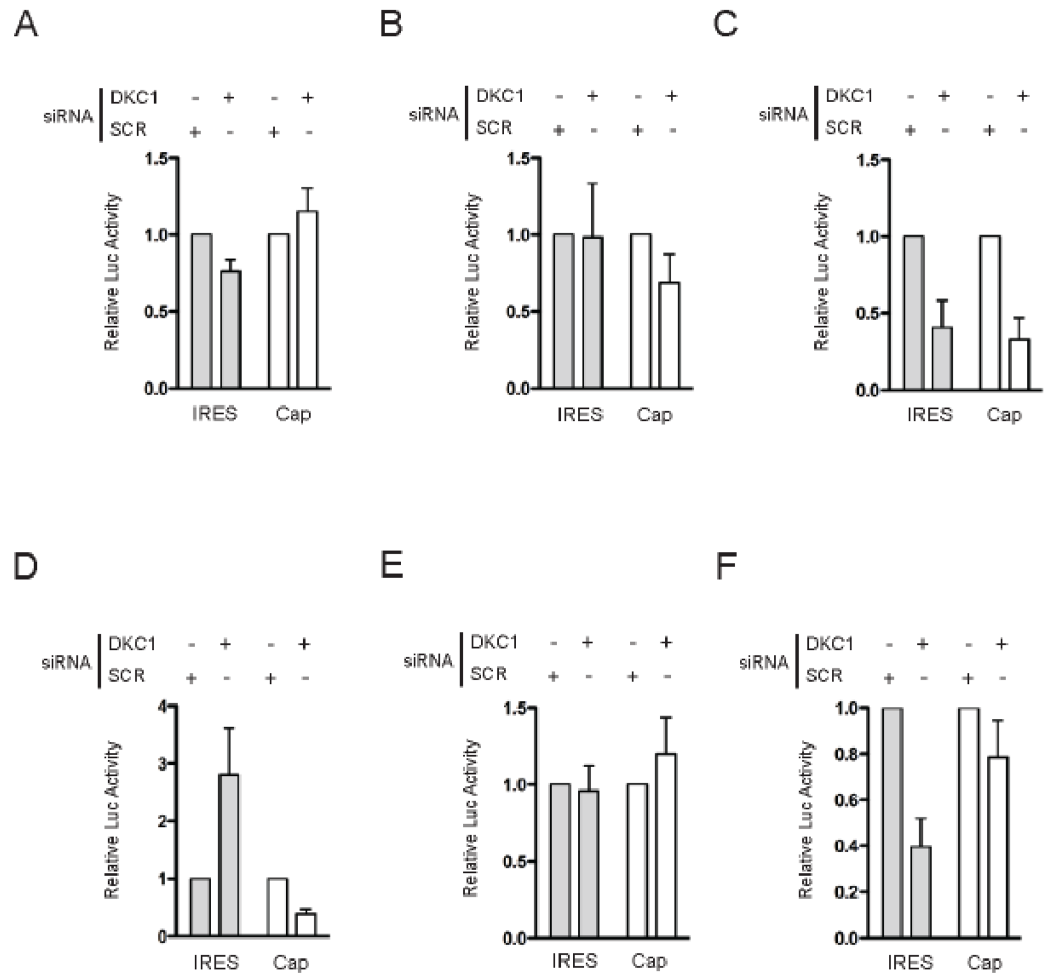

Figure S9. IRES mediated translation of different viral and cellular transcripts after DKC1 KD. The values of the IRES- and cap-mediated translation assessed by measuring the FLuc and RLuc activities in MCF-7 cells are reported. Cells were harvested 8 hours after the transfection with the bicistronic mRNA transcribed from viral pR-CrPV-IRES-F (A), pR-HCV-IRES-F (B) and pF-EMCV-IRES-R (C) and from cellular pR-HSP70-IRES-F (D), pR-c-MYC-IRES-F (E) and pR-p53-IRES-F (F). siRNA transfection was performed 96 hours before cell harvesting. Histograms represent means and SDs from three independent experiments.

Supplementary Figure S10

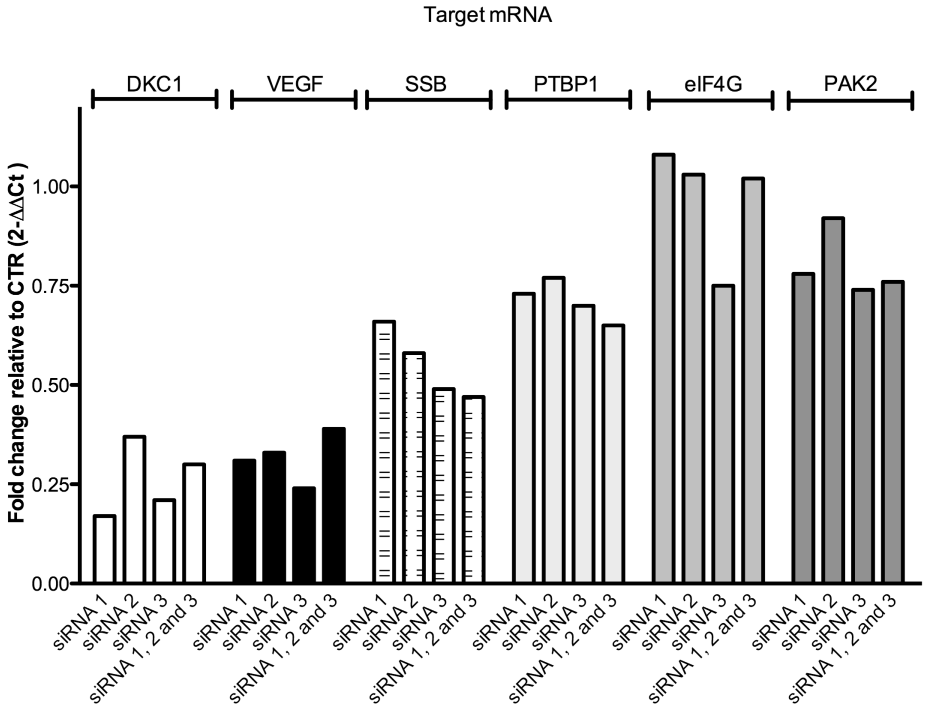

Supplementary Figure S10. Evaluation of the potential off-target effect of the DKC1 siRNAs used in the study on the expression of VEGF, SSB, PTBP1, eIF4G and PAK2.

The graph shows the results of a representative real time PCR experiment in which the potential off targed DKC1 KD on VEGF, EIF4G, Pak2, PTBP1, SSB/La was tested. siRNA 1 correspond to Invitrogen, catalog number HSS102781; siRNA 2 to HSS102782; siRNA 3 to HSS102785. Reaction was performed as described in the Materials and Methods section. Additional primers used for the assay were: SSB: 5'-GGGCCGGAACCTTAAAGATA 5'-TGACAGATTTTGGCCTCCA; PTBP1 5'-GACAAGAGCCGTGACTACACA 5'-TGCATACGGAGAGGCTGAGA; eIF4G 5'-TGGTGTTCAGTACGCCACAA 5'-GGCTAGGGTAGAAGTGCTGC; PAK2 5'-AAGGGGTTCAGCCAAAGAAT 5'-GATAACGGTTTGGCCAGTTTC.

Target mRNA expression values are shown as fold changes relative to control. Although different in terms of quantitative extent, the results obtained with each different single oligo was similar for each transcript tested (DKC1, VEGF, Pak2, SSB, PTBP1 - see Supplementary Figure S11) In our view, since it is very unlikely that different siRNA may have common off targets, these results strongly indicate that the results observed are due to the specific effect on DKC1 mRNA expression). Furthermore these results point out that the dowregulation of eIF4G induced by DKC1 KD (Supplementary Figure S8) is mainly post-transcriptional.

Supplementary Figure S11

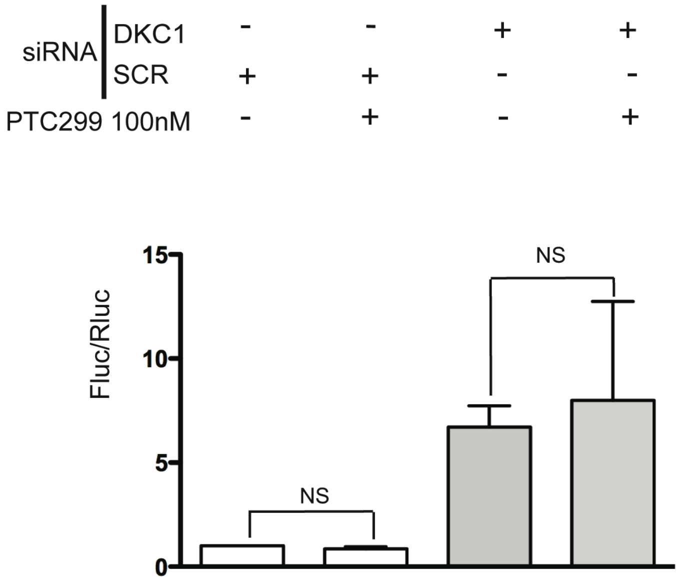

Figure S11. Effect of PTC299 on the IRES-mediated translation of an IRES-containing mRNA sensitive to DKC1 KD. IRES-mediated translation was assessed by measuring FLuc/RLuc activity ratio 8 hours after the transfection with the bicistronic mRNA transcribed from pRL-HSP70-IRES. MCF7 cells were treated with 100 nM PTC299 for 72 hours. siRNA transfection was performed 96 hours before cell harvesting. Histograms represent means and SDs from three independent experiments. NS=not significant.
